# SIGNET: transcriptome-wide causal inference for gene regulatory networks

**DOI:** 10.1038/s41598-023-46295-6

**Published:** 2023-11-08

**Authors:** Zhongli Jiang, Chen Chen, Zhenyu Xu, Xiaojian Wang, Min Zhang, Dabao Zhang

**Affiliations:** 1 Department of Statistics, Purdue University, West Lafayette, IN 47907 USA; 2 UCB Pharma, Brussels, 1070 Belgium; 3 ByteDance, Shanghai, 201107 China; 4 Department of Epidemiology and Biostatistics, University of California, Irvine, CA 92617 USA

**Keywords:** Gene regulatory networks, Machine learning, Software, Statistical methods

## Abstract

Gene regulation plays an important role in understanding the mechanisms of human biology and diseases. However, inferring causal relationships between all genes is challenging due to the large number of genes in the transcriptome. Here, we present SIGNET (Statistical Inference on Gene Regulatory Networks), a flexible software package that reveals networks of causal regulation between genes built upon large-scale transcriptomic and genotypic data at the population level. Like Mendelian randomization, SIGNET uses genotypic variants as natural instrumental variables to establish such causal relationships but constructs a transcriptome-wide gene regulatory network with high confidence. SIGNET makes such a computationally heavy task feasible by deploying a well-designed statistical algorithm over a parallel computing environment. It also provides a user-friendly interface allowing for parameter tuning, efficient parallel computing scheduling, interactive network visualization, and confirmatory results retrieval. The Open source SIGNET software is freely available (https://www.zstats.org/signet/).

## Introduction

Recently, gene regulatory networks (GRNs) have attracted increasing attention due to the availability of high-throughput gene expression data. GRNs can elucidate the disease mechanisms when cells are under dysregulation and greatly accelerate the wet lab experiments by precise predictions^1–3^. Many methods have been widely applied to construct GRNs based on gene co-expression^4, 5^. However, co-expression-based methods only infer association rather than direct causation. Hence the activator or repressor role of genes will remain ambiguous. On the other hand, unmeasured confounding variables and possible reverse causation challenge the utility of directed acyclic graphs for plausible causal interpretations between genes^6–8^.

A two-stage penalized least squares approach (2SPLS) has been developed to simultaneously conduct causal inference on all genes for their regulation with each other^9^. 2SPLS employs genotypic variants as instrumental variables, which also enables the practice of Mendelian randomization^10^. While Mendelian randomization can only establish a local causal relationship between a pair of genes, 2SPLS can construct a transcriptome-wide gene regulatory network^11^. This is a significant improvement, as it allows us to understand the complex interactions between genes in a more comprehensive way. 2SPLS is able to handle large amounts of transcriptomic and genotypic data by designing gene-based tasks of parallel computing at each of its two sequential stages^9^. This makes it possible to construct the transcriptome-wide gene regulatory networks that would otherwise be intractable.

We developed SIGNET, a handy and flexible platform for constructing transcriptome-wide GRNs. SIGNET takes advantage of 2SPLS and the computational power provided by clustered computers. SIGNET can be applied to transcriptomic and genotypic data for all tissues regardless of species. Driven purely by collected data, SIGNET applies the state-of-art 2SPLS as well as the greedy algorithm for clustering^12^ to automatically identify regulatory structures, prune for better accuracy, and report confidence on each constructed regulation. It also provides interactive visualization of the transcriptome-wide GRN and its subnetworks, and connects with public databases to provide validatory information on the identified causal relationships.

SIGENT is ready to apply to transcriptomic and genotypic data from The Cancer Genome Atlas (TCGA) project^13^ and the Genotype-Tissue Expression (GTEx)^14^ project. It also provides an interface to apply to user-preprocessed transcriptomic and genotypic data. We illustrated the use and capability of SIGNET by applying it to the Lung Adenocarcinoma (LUAD) data from TCGA and healthy lung tissue data from GTEx. With the LUAD data, we identified 4079 regulations for 4904 genes in each of the 1000 bootstrapped datasets. Similarly, with the healthy lung tissue data from GTEx, we identified 4301 regulations for 3603 genes. Many of these identified regulations have been reported in biological pathways with validated protein-protein interactions, however many others have not been reported before.

SIGNET is publicly available on GitHub with detailed documentation and example data for illustration. It allows users to quickly pick up the analysis and provides options for customization. A singularity container is provided for SIGNET, which can be used to run SIGNET on local servers or high-performance computing (HPC) clusters without having to install any additional software or libraries. This makes it easy for users to get started with SIGNET and reproduce their results.

## Results

### SIGNET workflow

**Figure 1:**
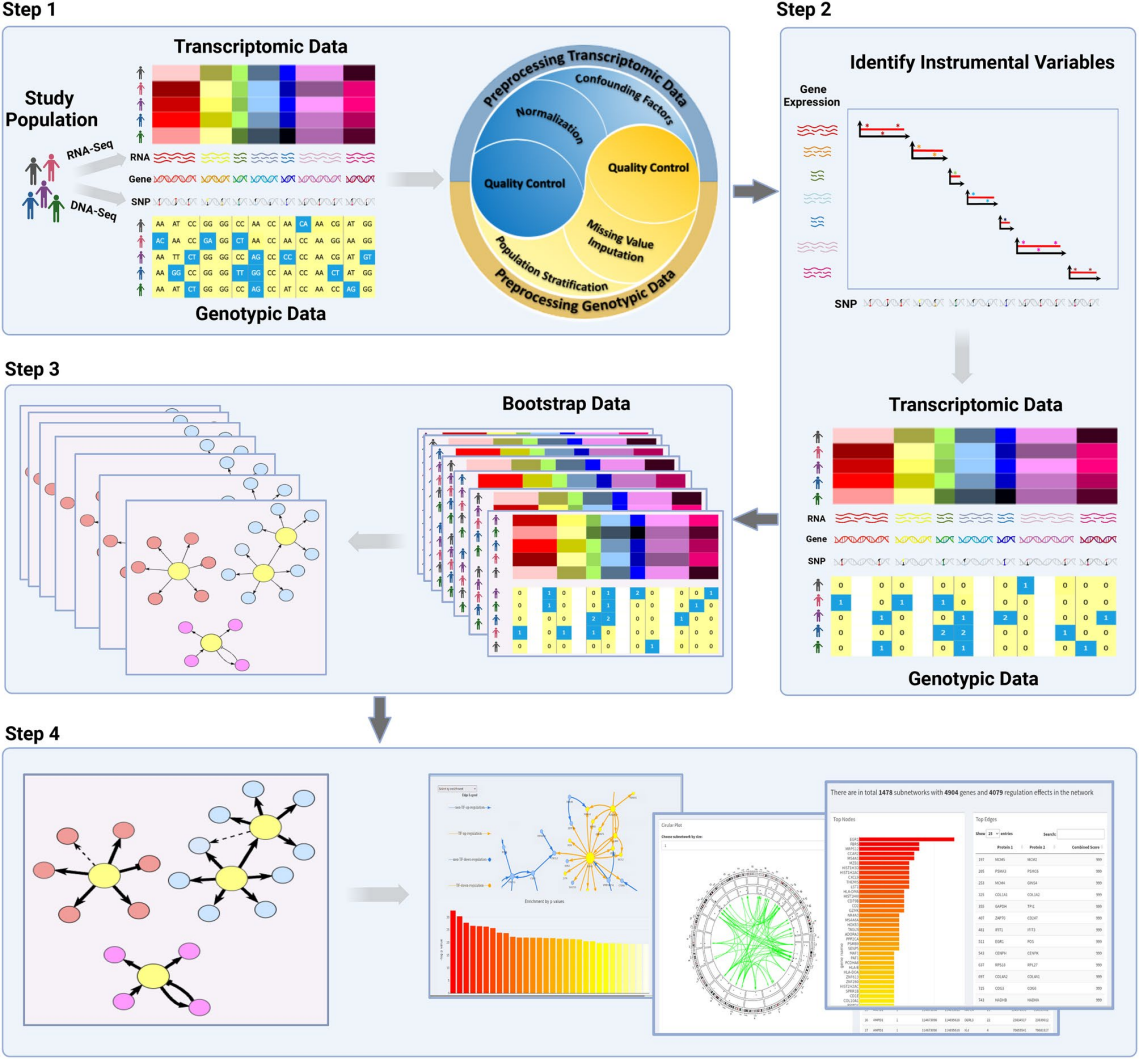
SIGNET workflow for gene regulatory network (GRN) construction. SIGNET takes four steps to conduct causal inference and construct a GRN from transcriptomic and genotypic data: 1. Preprocessing transcriptomic and genotypic data to ensure data quality; 2. Identifying genotypic instrumental variables; 3. Constructing a transcriptome-wide GRN via causal inference; 4. Visualizing subnetworks of the GRN and validating with public databases.

SIGNET has four main components, (i) preprocessing transcriptomic and genotypic data; (ii) identifying instrumental variables for each gene; (iii) causal inference of gene regulations; and (iv) visualizing constructed GRN with validatory information from public databases (Fig. 1). The four components can be easily integrated to set up a pipeline of constructing GRNs from transcriptomic and genotypic data. Each component is designed to work independently, allowing users to customize the pipeline with available functions in, e.g., R^15^, Bioconductor^16^, and PLINK^17^. Options are also provided at each step to fine-tune, e.g., cutoffs for filtering genotypic variants, and significance levels for identifying gene regulatory effects. In summary, SIGNET provides a flexible platform to meet users’ diverse demands in constructing transcriptome-wide GRNs with their own data or with data from TCGA and GTEx but with self-defined criteria. It is noteworthy that SIGNET can be applied to transcriptomic and genotypic data from any tissue of any taxonomy as long as both are preprocessed to pass quality control.

### Preprocessing transcriptomic and genotypic data

Both transcriptomic and genotypic data need to be processed before they can be used to construct GRNs. This processing includes removing low-quality data, imputing missing values, and correcting for confounding effects. There are well-established protocols and pipelines for processing each type of data^18, 19^. SIGNET streamlines the data preprocessing procedures for transcriptomic and genotypic data provided by TCGA and GTEx^14, 20, 21^. It provides separate functions for each type of data, making it easy to process data from either source.

For transcriptomic data, SIGNET filters out genes with low reads to improve the statistical power. It handles the heteroscedasticity in the count data and normalizes the data by taking account of the library sizes. SIGNET transforms the transcriptional abundance with a base-2 logarithm for the downstream analysis, using the variance stabilizing transformation (VST)^20^ for TCGA data and TMM^21^ for GTEx data, respectively. SIGNET allows users to select only protein-coding genes to work in the downstream analysis. As confounding factors may lead to spurious association and result in false regulation, SIGNET provides functions to adjust for confounding effects from race, gender, and possible population stratification. An interactive interface is provided to help identify necessary principal components to account for the genetic differences in the population.

For genotypic data, SIGNET provides a function for preprocessing TCGA data. This function assembles procedures such as quality control using PLINK^17^, removal of genotypic variants and samples with high missing rates, and disposal of single nucleotide polymorphisms (SNPs) discordant with Hardy Weinberg equilibrium (HWE). SIGNET streamlines the SNP imputation procedure in parallel using IMPUTE2^22^. This significantly speeds up the process by simultaneously imputing missing values of multiple genetic regions. SIGNET also provides a function that combines and streamlines the procedures in the GTEx pipeline^14^. This function starts with the phased genotypic data which are directly available at dbGaP^23^ and have passed through quality control.

### Identifying genotypic instrumental variables

Possible confounding factors and reverse causation make it challenging to conduct causal inference in observational studies. For a successful inference, we need instrumental variables (IVs) which (i) are associated with the exposure; (ii) are independent of the confounders of both exposure and outcome; (iii) affect the outcome only through the exposure^24, 25^. As shown in Mendelian randomization, genotypic variants in a gene’s genetic region, that is, the gene’s cis genotypic variants, have the random assignment nature during meiosis, so they have the aforementioned properties and serve naturally as instrumental variables for their host genes. SIGNET can detect significant cis-acting genotypic variants, i.e., genetic variants in a gene’s genetic region, for each gene at a prespecified significance level (0.05 by default) and may use multiple cis-acting genotypic variants as instrumental variables.

### Revealing transcriptome-wide gene regulation

SIGNET implements 2SPLS^9^, which takes advantage of IVs identified in the previous stage to infers transcriptome-wide causal regulatory networks. Unlike many methods constructing directed acyclic graphs (DAGs) to decipher causality in gene regulations, SIGNET builds non-recursive yet directed cyclic graphs (DCGs) to realistically describe causal regulations between genes. This is because DCGs can capture reciprocal regulation between genes or regulatory feedback loops among a group of genes. Statistically, for any gene with its cis genotypic variants as instrumental variables, the rank condition^26^, serving as a necessary and sufficient condition for uniquely estimating parameters in a system of equations, assures that SIGNET can systematically explore and identify all causal effects on other genes.

With the parallel nature of 2SPLS, SIGNET is computationally fast in constructing a transcriptome-wide GRN from a set of transcriptomic and genotypic data. This allows SIGNET to bootstrap the original dataset, construct a causal regulatory network for each bootstrap dataset, and aggregate all networks to infer a transcriptome-wide GRN with desired confidence (Fig. 1).

SIGNET can estimate the total execution time and automatically optimize the allocation of available computing resources for distributed computing over server clusters. It can submit jobs and collect the results on any high performance computing (HPC) cluster with SLURM (Simple Linux Utility for Resource Management) Workload Manager^27^. With multi-nodes with multi-cores in an HPC cluster, SIGNET can construct a transcriptome-wide GRN from hundreds of samples in a day. With two stages of parallel computing in 2SPLS, SIGNET instantly summarizes the results upon the completion of the first stage and submits the jobs for the second stage. It also allows users to customize the parallel computing at each of the two stages.

SIGNET reports its constructed causal network using an adjacency matrix, where each entry encodes the confidence of the corresponding regulation. With a customized confidence level, the whole network may be broken down into disconnected subnetworks. SIGNET provides functions to output and further inspect these subnetworks. These subnetworks can be saved in files with various formats, allowing users to conduct downstream analysis using other packages such as STRING^28^, Cytoscape^29^, and Ingenuity Pathway Analysis (IPA)^30^.

### Visualizing gene regulatory networks

The causal network constructed by SIGNET may involve thousands of genes and hence tens of thousands of possible regulations. The huge size of such networks makes it challenging to visualize and interpret. To address this challenge, SIGNET provides a web-based interactive interface that is developed upon R package Shiny^31^ and allows users to explore the rich results in constructed networks and incorporate biological interpretation from STRING^28^. With an adjacency matrix recapitulating the bootstrap results from the above network construction, users may customize the confidence level and take SIGNET to summarize the constructed network, e.g., reporting the numbers of genes and regulations involved as well as hub genes and pivotal interactions. Such hub genes and pivotal interactions together with the underlying sub-networks may direct users to the most relevant protein complex for further investigation. For example, pertinent confirmatory information may be obtained from the STRING database^28^ which reports protein-protein interaction (PPI) scores, indicating confidence shown in biochemical experiments, co-expressions, and other databases.

SIGNET allows users to search for genes of interest and identify their connections with other genes. SIGNET can break down the constructed networks into subnetworks based on network modularity^32^. By characterizing the divisibility of a network, SIGNET can identify densely connected communities within the network, where regulations are much denser than interactions between subnetworks. SIGNET utilizes STRING to extract enriched pathways, which are subsequently employed to partition the network. In fact, SIGNET provides a bar plot of these enriched pathways shown in decreasing order of *p* values. Genes can be selectively highlighted for investigation of their specific function, facilitating the study of their enriched effect. SIGNET also accentuates transcript factors for their important roles in the network. With the interactive interface provided by SIGNET, users can select two connected genes and check for pertinent confirmatory information in other databases. The interactive plots on GRN are also accessible in a portable HTML format, allowing for effortless sharing and dissemination of the plots among fellow researchers (Supplementary Files 1, 2).

SIGNET is able to complete all aforementioned interactive visualization and clustering on subnetwork community structures efficiently. It provides an R shiny-based interactive application for easy access. The visualization functions provided by SIGNET can also be applied to networks constructed elsewhere, with adaptability to various genome assemblies and species. SIGNET can generate multiple portable results, making it flexible to conduct downstream analysis using other packages. Users may integrate the visualization function of SIGNET and other databases to generate numerous innovative biological hypotheses for further study.

### Transcriptome-wide GRN for healthy lung tissues

**Figure 2:**
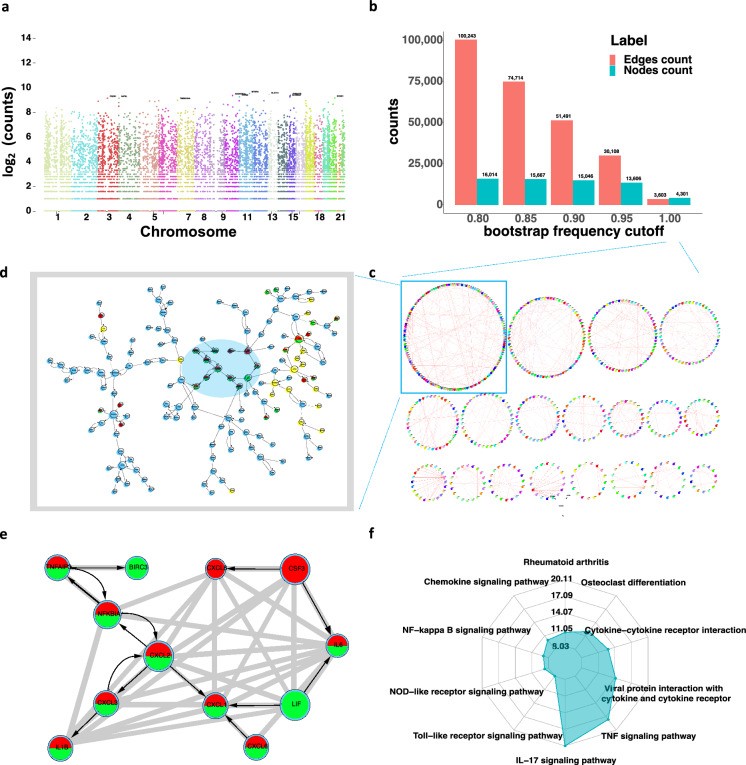
Results of analyzing the GTEx data for healthy lung tissues. (**a**) Manhattan plot of numbers of IVs across all chromosomes. (**b**) Histogram of numbers of edges and nodes with respect to different bootstrap frequency cutoffs. (**c**) Circular plots of the largest subnetworks in Cytoscape, with darker color indicating the larger size of regulatory effects. (**d**) The largest subnetwork, with transcription factors highlighted in yellow and node sizes proportional to node degrees. (**e**) Highlight of gene regulations shaded in d with gray connections verified by STRING, which also identifies genes in red and green enriched in IL-17 and TNF signaling pathways, respectively. (**f**) Radar plot of the ten KEGG pathways in which the subnetwork in d is enriched the most, with IL-17 and TNF signaling pathways as the top two.

We applied SIGNET to construct the transcriptome-wide GRN for healthy lung tissues using transcriptomic data from lung tissues and genotypic data from the blood of 482 healthy individuals in the GTEx study^14^. Out of a total of 16,761 protein-coding genes passing the quality control, 10,965 genes were identified with unique IVs, consisting of 279,504 SNPs or SNP regions (Fig. 2a). SIGNET detected 4301 gene regulations involving 3603 genes, comprising 1325 subnetworks in each of 1000 bootstrap datasets, and 30,108 gene regulations involving 13,606 genes in over $95\%$ of these bootstrap datasets (Fig. 2b, Supplementary Table 5 for complete listing).

We investigated the GRN detected in every bootstrap dataset, and identified the largest subnetwork shown in Fig. 2d, which consists of 145 genes including 23 transcription factors. Validation in STRING^33^ shows that this set of genes is enriched in 19 human KEGG pathways ($p\le 10^{-5}$) with the top ten shown in Fig. 2f, including 21 genes found in the IL-17 signaling pathway ($p=7.04 \times 10^{-24}$) and 20 genes in the TNF signaling pathway ($p=6.49 \times 10^{-21}$), both of which play an important role in the immune response (Supplementary Table 6). As highlighted in Fig. 2d,e, STRING also shows rich connections between genes, evidenced via text mining, experiments, database, and co-expression with a score over 0.8. However, our constructed GRN further reveals the causal regulation in comparison to mere interaction. The GRN constructed on LUAD also finds significant enrichment in IL-17 signaling pathway and TNF signaling pathway on its fifth largest subnetworks (Supplementary Note, Supplementary Fig. 1).

**Figure 3:**
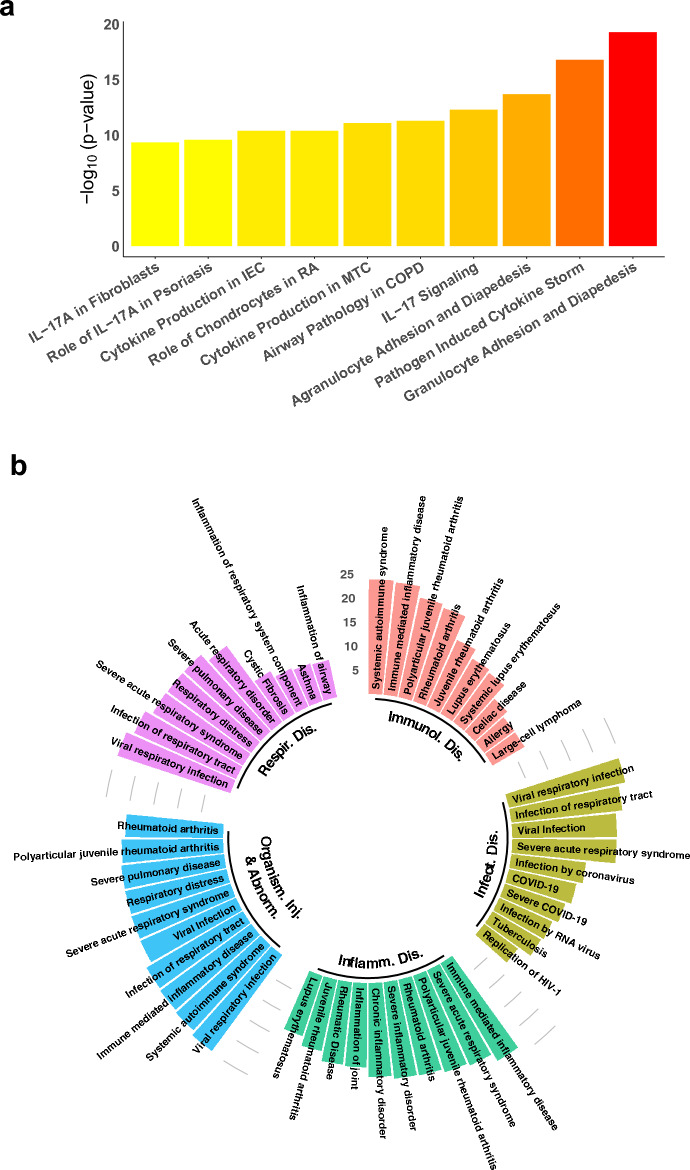
IPA validation of the top subnetwork constructed for healthy lung tissues. (**a**) Top ten significant Ingenuity canonical pathways, with IEC abbreviated for Intestinal Epithelial Cells, RA for Rheumatoid Arthritis, MTC for Macrophages and T Helper Cells, and COPD for Chronic Obstructive Pulmonary Disease. (**b**) The five most significant types of diseases and functions identified by IPA, with each type shown as the top ten significant diseases/functions.

We also validated the same set of genes using IPA^30^ but restricted to human lung tissues. We identified 35 Ingenuity canonical pathways enriched with these genes ($p\le 10^{-5}$), with top ten pathways shown in Fig. 3a (Supplementary Table 7 for complete listing). Note that half of the top ten pathways are related to IL-17, as both pathways on cytokine production are on the differential regulation of cytokine production between IL-17A and IL-17F. IPA also reports that this set of genes is significantly associated with 55 types of diseases and functions ($p\le 10^{-5}$), with top five shown in Fig. 3b (Supplementary Table 8 for complete listing). In fact, there are 37 genes in respiratory disease, 74 genes in infectious disease, and 126 genes associated with organismal injury and abnormalities. In each of these three types, viral respiratory infection is the most significant disease involving 28 genes from the subnetwork ($p=2.50\times 10^{-25}$). Furthermore, this subnetwork has 30 genes associated with COVID-19 ($p=5.72\times 10^{-15}$) and 17 with severe COVID-19 ($p=8.19\times 10^{-14}$). Akin to the result for healthy lung tissues, the fifth-largest subnetwork in GRN constructed using LUAD data is also significantly enriched with COVID-19 (Supplementary Note, Supplementary Fig. 2).

## Discussion

SIGNET is an open-source software that can construct causal networks of gene regulation purely based on user-provided transcriptomic and genotypic data, incorporating biological information on the genome. SIGNET employs genotypic variants as natural instrumental variables, making it feasible for causal inference. Our method builds upon a structural graph describing regulatory causality between all genes and intends to construct a transcriptome-wide GRN, rather than local causal inference on a single exposure-outcome pair as traditional Mendelian randomization does. Providing a comprehensive map of gene interactions, transcriptome-wide GRNs can help us understand cellular mechanisms and disease pathways^1, 34^, as well as accelerating drug discovery^35^ and developing broad-based therapeutics of different diseases^36^.

Although the task of transcriptome-wide causal inference is formidable, SIGNET implements 2SPLS^9^, which innovatively employs a penalized limited-information method to construct causal networks in two sequential stages, with each stage parallelly conducting computation on a batch of genes. This makes it feasible to construct transcriptome-wide GRN in HPC. For example, in constructing networks for 1000 datasets bootstrapped from the healthy lung dataset, SIGNET took 5.7 h to complete 10,965,000 tasks (in 3000 parallel jobs) at the first stage and 16,761,000 tasks (in 7000 parallel jobs) at the second stage using Purdue HPC with 448 nodes of Two Rome CPUs (2.0GHz), each having 128 cores. Without parallel computing, it would take more than 3 years to complete. Moreover, SIGNET can set up parallel tasks adaptable to available cores, memories, and wall time of HPC, alleviating the burden for users unfamiliar with parallel computing.

2SPLS is built upon the assumption of linear causal systems and well-developed identification results on such systems. The recent development of ensemble models and deep learning methods makes it appealing to explore nonlinear causal systems, e.g., constructing GRNs without the linear assumption. However, such an endeavour is still challenged by the model identification issue, i.e., when and how we are assured that a constructed relationship is truly causal. Furthermore, building ensemble models implies more computational burdens, and deep learning methods also require a sufficient amount of data.

SIGNET is available with a Singularity container, which includes all the required software and packages. The container is user-friendly and can save users a huge amount of time setting up the required computational environment. SIGNET is also a flexible command-line tool that allows users to adjust multiple parameters to customize their analysis. Additionally, SIGNET provides independent functional units that advanced users can easily modify and integrate with their own analyses. We also provide the example data and a detailed document with step-by-step instructions.

## Methods

### Transcriptomic data preprocessing

SIGNET sets up the preprocessing procedures for transcriptomic data following two studies, i.e., GTEx and TCGA, and provides template functions for each study^14, 20, 21^. The function for preprocessing GTEx data assumes input files including gene count data and gene TPM (transcript per million), directly from the GTEx portal (https://gtexportal.org/home/datasets). The function for preprocessing TCGA data takes the log-transformed HTSeq gene count data, available at UCSC Xena^37^, and transforms them back to the original gene counts.

SIGNET follows the GTEx pipeline^14^ to conduct the quality control for GTEx data. It selects genes with TPM greater than 0.1 in at least 20% of the samples and at least six reads in at least 20% of samples. For TCGA data, SIGNET filters out genes with total counts less than 2.5 million or missing in more than $80\%$ of the samples^38^. Both types of transcriptomic data are normalized via base-2 logarithm transformation, with GTEx data normalized via the TMM method^21^ available in the edgeR package^39^ and TCGA data normalized via the variance stabilizing transformation available in the DESeq2 package^40^.

### Genotypic data preprocessing

SIGNET streamlines the preprocessing procedure for genotypic data from both GTEx and TCGA^14, 41, 42^, and provides corresponding functions for each study. The function for preprocessing GTEx data assumes phased genotypic data after quality control, which are directly available at dbGaP (https://www.ncbi.nlm.nih.gov/gap/). It filters out genetic variants with the total counts of minor alleles across samples of fewer than five. The function for preprocessing TCGA data takes the input of genotypic data in PLINK file format^17^, which can be converted from the BAM files available in the GDC data portal (https://portal.gdc.cancer.gov/). It follows the GDC bioinformatics pipeline^42^. The more detailed data information and data preprocessing procedures, which include conversion from whole-exome sequencing BAM files to PLINK format, are available in Supplementary Note.

SIGNET excludes samples and genetic variants with high missing rates. By default, it excludes samples with a missing rate of more than 10% across genetic variants and genetic variants with a missing rate of more than 10% across samples. It then filters out genetic variants discordant with the Hardy-Weinberg equilibrium, tested via PLINK with a *p*-value cutoff at 0.0001 by default. The missing values are imputed via IMPUTE2^22^ with 1000 Genomes Phase 3 as the reference genome^43^. This may be time-consuming, so SIGNET speeds up the process by simultaneously imputing multiple genetic regions, e.g., each region with $5 \times 10^6$ base pairs by default. For variants missing in the reference genome, SIGNET imputes their missing values with the major alleles.

### Adjusting for confounding factors

SIGNET uses linear regression to remove the effects of potential confounding factors from the gene expression data for subsequent causal inference. It provides separate functions for GTEx and TCGA because there are different factors available in the two studies. Specifically, SIGNET removes the confounding effects of sex, sequencing platform (Illumina Hiseq2000 or Illumina HiseqX), and library construction protocol (PCR based or PCR free) from gene expression in GTEx data^14^, but only races and sex from gene expression in TCGA data^13^.

Population stratification via principal components (PCs) of genotypic data is an important step in local association studies. PCs can be used to control the confounding effects of other genetic variants^44^. SIGNET removes the effects of top PCs (top three PCs by default) from the gene expression data before identifying IVs. This is because SIGNET is conducting local association studies for IVs. On the other hand, the gene expression data used for the transcriptome-wide causal inference are not adjusted for these PCs. This is because the transcriptome-wide causal inference is designed to identify global patterns of gene regulation, and adjusting for PCs would remove some of this information.

### Genotypic instrumental variables identification

Similar to Mendelian randomization, SIGNET leverages available genetic polymorphisms in a gene’s genetic region as its potential IVs. By default, SIGNET scans the cis-acting genetic polymorphisms located within both the start and end sites of genes, as well as 1000 base pairs upstream and downstream of these regions. SIGNET then categorize the polymorphisms according to their minor allele frequency (MAF): common variants with MAF no less than 0.05, low-MAF variants with MAF no less than 0.01, and rare variants with MAF less than 0.01. Common variants are scanned directly for their qualification of serving as IVs. Both low-MAF and rare variants go through a data-adaptive burden test, aSum test^45^, which aggregately constructs possible IVs to avoid loss of power caused by opposite effects of variants.

SIGNET provides a platform to identify genotypic IVs in parallel by conducting association studies of expression traits of many genes on their own genetic regions. In particular, SIGNET implements the aSum test as a permutation test, which is computationally intensive. SIGNET provides a simple portal that automatically divides the whole genome into separate regions and uses parallel computing to efficiently conduct the related tests. A full list of identified IVs for healthy lung tissue and LUAD is available in Supplementary Tables 4 and 9. In the case of multiple cis-variants detected for a gene, SIGNET will select the top three which are decoupled with pairwise correlation under 0.3 by default.

### Causal inference model

We focus on a linear system with *p* genes and *q* genotypic variants, which are observed in a sample of *n* observations. Let $\textbf{Y}_{n\times p} = (\textbf{Y}_1, \ldots , \textbf{Y}_p)$ and $\textbf{X}_{n\times q} = (\textbf{X}_1, \ldots , \textbf{X}_q)$ denote the gene expression and genotypic data, respectively. The gene-gene regulations and the genetic effects of variants can be described by the following structural equations,

1 $$\begin{aligned} \textbf{Y}=\textbf{Y}\varvec{\Gamma } + \textbf{X}\varvec{\Psi } + \varvec{\epsilon } \end{aligned}$$

where $\varvec{\Gamma }$ is a $p\times p$ matrix with all diagonal elements equal to zero and its non-diagonal elements indicating regulatory effects; $\varvec{\Psi }$ is a $q\times p$ matrix with the majority of elements known to be zero and its non-zero components indicating cis effects of corresponding variants; ${\varvec{\epsilon }}$ is an $n\times p$ matrix of disturbance errors, independent of $\textbf{X}$.

With tens of thousands of genes and even more genotypic variants available in a system, directly maximizing the likelihood for model (1) is a formidable task. Instead, when we are only concerned with how all other genes regulate the *k*-th gene, we can deal with the following limited information model,

2 $$\begin{aligned} \left\{ \begin{array}{l} \textbf{Y}_k = \textbf{Y}_{-k}\varvec{\gamma }_k + \textbf{X}\varvec{\psi }_k+\varvec{\epsilon }_k,\\ \textbf{Y}_{-k} = \textbf{X}\mathbf {\varvec{\pi }}_{-k} + \mathbf {\varvec{\xi }}_{-k}. \end{array}\right. \end{aligned}$$

Note that the first part of the above model is simply the *k*-th structural equation in the model (1), so $\textbf{Y}_{-k}$ refers to $\textbf{Y}$ excluding the *k*-th column, i.e., the expression of all genes except gene *k*; $\varvec{\gamma }_k$ refers to the *k*-th column of $\varvec{\Gamma }$ excluding the diagonal zero, indicating all other genes’ regulatory effects on gene *k*; $\varvec{\psi }_k$ (cis effects of variants of gene *k*) and $\varvec{\epsilon }_k$ refer to the *k*-th columns of $\varvec{\Psi }$ and $\varvec{\epsilon }$ respectively. The second part of the model (2) is from the reduced model derived from model (1), and is necessary for estimating $\varvec{\gamma }_k$ via the model (2). Therefore, the limited information model (2) allows a multiple Mendelian randomization to identify regulatory genes for gene *k*.

### Multiple mendelian randomization

The success of the multiple Mendelian randomization on the model (2) relies on available IVs for each gene included in $\textbf{Y}_{-k}$. With a large number of genes investigated simultaneously for their regulatory effects on a single gene, SIGNET follows 2SPLS^9^ to predict the expression levels of each potential regulatory gene using all available IVs, which are first screened via the iterative sure independence screening method (ISIS)^46^. Such a prediction, say $\hat{\textbf{Y}}_j$ for $\textbf{Y}_j$ of each gene *j*, is optimized by ridge regression^47^ combined with the generalized cross validation method (GCV)^48^ selecting the best tuning parameters.

Because the majority of elements in $\varvec{\psi }_k$ are known to be zero, we denote the set with indices with nonzero elements as $\mathscr {S}_k$. Then

$$\begin{aligned} \textbf{X}\varvec{\psi }_k = \textbf{X}_{\mathscr {S}_k}\varvec{\psi }_{k,\mathscr {S}_k}. \end{aligned}$$

Further, we denote an orthogonal projection matrix for the column space of $\textbf{X}_{\mathscr {S}_k}$ as

$$\begin{aligned} \textbf{H}_k = I_n-\textbf{X}_{\mathscr {S}_k}\left( \textbf{X}_{\mathscr {S}_k}^T \textbf{X}_{\mathscr {S}_k}\right) ^{-1} \textbf{X}_{\mathscr {S}_k}^T, \end{aligned}$$

which is computational feasible and involves only low-dimensional matrices as $\mathscr {S}_k$ is a small set. Note that, if gene *k* does not have any IVs, $\textbf{H}_k$ is simply an identity matrix.

With predicted $\hat{\textbf{Y}}_{-k}$ for $\textbf{Y}_{-k}$, we can apply adaptive LASSO^49^ to the following high-dimensional regression,

$$\begin{aligned} \textbf{H}_k \textbf{Y}_k = \textbf{H}_k \hat{\textbf{Y}}_{-k} \varvec{\gamma }_k + \varvec{\zeta }_k, \end{aligned}$$

where $\varvec{\zeta }_k$ is the error term and $\varvec{\gamma }_k$ corresponds to the same potential regulatory effects in model (2). Nonzero elements in the estimated $\varvec{\gamma }_k$ indicate that gene *k* is causally regulated by the corresponding gene. It has been proved that the estimated regulatory effects have well-bounded errors and identified gene regulatory causality is statistically consistent with the underlying gene regulatory network^9^.

### Transcriptome-wide causal inference

The above multiple Mendelian randomization will identify all regulatory genes and estimate relevant regulatory effects for each gene, say gene *k*, in two stages: (1) predicting $\textbf{Y}_{-k}$ with $\hat{\textbf{Y}}_{-k}$; (2) identifying and estimating regulatory effects by regressing $\textbf{H}_k \textbf{Y}_k$ against $\textbf{H}_k \hat{\textbf{Y}}_{-k}$. Since $\textbf{Y}_{-k}$ is a subset of $\textbf{Y}$, SIGNET implements the algorithm by first predicting each individual $\textbf{Y}_{k}$, $k=1, 2, \ldots , p$. Therefore, both stages can be computed in parallel, which allows high performance computing clusters to quickly conduct transcriptome-wide causal inference.

SIGNET constructs model (1) to depict transcriptome-wide causal inference of gene regulation. In the first stage, SIGNET pools together genotypic IVs over the whole genome and take them to predict the expression values of each gene. SIGNET applies the ridge regression function available in R package MASS^50^ for the prediction purpose, with the tuning parameter optimized by GCV. SIGNET uses R package parcor^51^ to implement adaptive lasso. At the completion of construction, SIGNET outputs the results as a sparse adjacency matrix, with each (*i*, *j*)-th component including the regulatory effect of *j*-th gene on *i*-th gene.

### Bootstrapping for confidence of gene regulatory effects

The parallel scalability of 2SPLS makes it possible to employ the bootstrap method to evaluate the reliability of each constructed regulation. This is usually a challenging task because of the enormous parameters involved in a transcriptome-wide GRN. For each bootstrap dataset, we will apply 2SPLS^9^ to conduct transcriptome-wide causal inference of gene regulation. The regulatory effects are stored in matrix $C^{(b)}$ for the *b*-th bootstrap dataset, with its component $C_{ij}^{(b)}$ denoting the regulatory effect of gene *j* on gene *i*. The corresponding transcriptome-wide GRN can be described by an adjacency matrix $A^{(b)}$, with its component $A^{(b)}_{ij}$ defined as $A^{(b)}_{ij}=1$ if $C^{(b)}_{ij}\ne 0$ and $A^{(b)}_{ij}=0$ if $C^{(b)}_{ij}=0$. With a total of *B* networks constructed from *B* bootstrap datasets, SIGNET averages across all adjacency matrices componentwise for the frequencies of regulations identified between each pair of genes,

3 $$\begin{aligned} \bar{A} = \frac{1}{B}\sum \limits _{b=1}^B A^{(b)}. \end{aligned}$$

### Automated parallel computing

SIGNET automates the divide-and-combine steps for parallel computing. For each stage involving parallel computing, SIGNET randomly selects 10 genes from the input data set and runs with them to evaluate the computational burden. Specifically, SIGNET records the maximum running time and memory consumption for these genes and employ this information to determine the optimal number of genes for each batch of the task. SIGNET then configures batch scripts and submits them via the SLURM scheduler. Upon completion of the jobs, SIGNET collects the results, which include coefficient matrices and adjacency matrices as mentioned in the previous section, and automatically summarizes the regulatory relationships of all genes identified from all bootstrap datasets.

### Partitioning GRN into subnetworks

With the averaged adjacency matrix $\bar{A}$ calculated in (3), we can visualize the transcriptome-wide GRN, including gene regulations identified over a pre-specified bootstrap frequency. For example, for a frequency cutoff *p*, we can derive a directed graph (*V*, *E*) describing the GRN, with *V* including all the involved genes and *E* calculated with its component

$$\begin{aligned} E_{ij} = I[\bar{A}_{ij}\ge p], \end{aligned}$$

where $I[\cdot ]$ is an indicator function.

For each gene *i*, SIGNET can calculate its total degree as

$$\begin{aligned} d(i) = \sum \limits _{j\in V: j\ne i} (E_{ij} + E_{ji}), \end{aligned}$$

which counts the number of genes regulating and regulated by gene *i*. With a partition $\mathscr {D}$ of the graph (*V*, *E*) into certain subgraphs, we denote $g_{\mathscr {D}}(i)$ as the subgraph including gene *i*, and $\delta _{\mathscr {D}}(\cdot ,\cdot )$ an indicator function on whether two genes belong to the same subgraph, i.e., $\delta _{\mathscr {D}}(i, j) = I[g_{\mathscr {D}}(i)=g_{\mathscr {D}}(j)]$. With *N* the total number of regulations in the graph (*V*, *E*), the modularity, under partition $\mathscr {D}$, is calculated as,

$$\begin{aligned} Q(\mathscr {D}) = \frac{1}{2N} \sum \limits _{(i,j)}\left( (E_{ij} + E_{ji})-\frac{d(i)\times d(j)}{2N}\right) \times \delta _{\mathscr {D}}(i,j). \end{aligned}$$

It measures the goodness of partition $\mathscr {D}$ in defining subnetworks of our constructed GRN by quantifying the within-subnetwork regulations. SIGNET maximizes this modularity to obtain the optimal partition by using the fast greedy modularity optimization algorithm^12^, which is implemented in the R packages igraph^52^.

### Supplementary Information

Supplementary Information 1.

Supplementary Information 2.

Supplementary Information 3.

Supplementary Information 4.

Supplementary Information 5.

Supplementary Information 6.

Supplementary Information 7.

Supplementary Information 8.

Supplementary Information 9.

Supplementary Information 10.

Supplementary Information 11.

Supplementary Information 12.

Supplementary Information 13.

## Data Availability

The results produced here are in whole or part based upon data generated by the TCGA Research Network (https://www.cancer.gov/tcga). The gene count data could be downloaded from UCSC Xena (https://xenabrowser.net/datapages/) and genotypic data are retrieved from the GDC portal (https://portal.gdc.cancer.gov/). For the GTEX project, the gene count data was obtained from the GTEx portal (https://www.gtexportal.org/home/datasets). Genotypic data are obtained from dbGaP with accession number phs000424.v8.p2 on July 23, 2019.
