# Supplementary material for: SIGNET: transcriptome-wide causal inference for gene regulatory networks
Source: Sci Rep. 2023 Nov 8;13:19371. doi: 10.1038/s41598-023-46295-6 (PMC10632394; doi:10.1038/s41598-023-46295-6)
Supplement: Supplementary file 3 — Supplementary Information 3. [file 41598_2023_46295_MOESM3_ESM.pdf]

Supporting Information for

SIGNET: Transcriptome-Wide Causal Inference for Gene Regulatory Networks

This PDF file includes:

Supplementary Notes
Supplementary Figures 1-2
Supplementary Tables 1-13
Supplementary Files 1-2
Supplementary Codes 1-2

Supplementary Notes

S.1 Transcriptome-wide Gene Regulation for Healthy Lung Tissues

S.1.1 GTEx Data for Healthy Lung Tissues

The Genotype-Tissue Expression (GTEx) dataset[1] includes both transcriptomic and genotypic information of 838 postmortem donors, encompassing 49 different tissues. Phased genotypic data of all donors across the 49 tissues were obtained from dbGaP with accession number phs000424.v8.p2 in a single VCF file. The VCF file was first converted to PLINK format files using PLINK [2], consisting of 43,066,422 SNPs for each sample. The gene count data were downloaded from the GTEx portal (https://www.gtexportal.org/home/datasets), containing a total of 56,200 genes.

S.1.2 Data Preprocessing, Causal Inference, and Codes

Transcriptomic and genotypic data were separately preprocessed as described in the main text under "**Preprocessing Transcriptomic and Genotypic Data**". SIGNET kept the samples with both germline genotypic data collected from blood and transcriptomic data collected from lung tissues, resulting in a total of 482 matched samples. After quality control, 482 samples with 26,069 genes and 12,980,438 SNPs were then used in SIGNET for causal inference of transcriptome-wide GRN. The codes are shown in **Supplementary Codes 1** with SIGNET installed and configured according to **Supplementary Table 1**, and GTEx data was organized according to **Supplementary Table 2**.

S.1.3 Results and Annotation of the Transcriptome-wide GRN

The results are described in the main text under "**Transcriptome-wide GRN for Healthy Lung Tissues**", with some annotations shown in **Fig. 2** and **Fig. 3**.

S.2 Transcriptome-wide Gene Regulation for Lung Adenocarcinoma

S.2.1 TCGA Data for Lung Adenocarcinoma

The Cancer Genome Atlas (TCGA) cohort [3] comprises more than 20, 000 primary cancer and corresponding normal samples, encompassing 33 distinct types of cancer. We retrieved whole-exome sequencing data of Lung Adenocarcinoma (LUAD) in BAM files from the GDC data portal (https://portal.gdc.cancer.gov/) and aligned them to the GRCh38.d1.vd1 human reference genome via Burrows-Wheeler Aligner[4], following the GDC bioinformatics pipeline. Germline variants were called using Strelka2 [5] and output genomic VCF files. The VCF files were merged and genotyped using gVCF Genotyper coming with Strelka2, after which we converted the VCF files to PLINK format files using PLINK. Gene count data for LUAD were downloaded from UCSC Xena (https://xenabrowser.net/datapages/), containing a total of 60,489 identifiers.

S.2.2 Data Preprocessing, Causal Inference, and Codes

Transcriptomic and genotypic data were separately preprocessed as described in the main text under "**Preprocessing Transcriptomic and Genotypic Data**". SIGNET kept the 528 tumor samples of LUAD with both germline genotypic and transcriptomic data. After quality control, 528 samples with 17,305 genes and 21,302,487 SNPs were then used in SIGNET for causal inference of a transcriptome-wide GRN. The codes are shown in **Supplementary Codes 2** with SIGNET installed and configured according to **Supplementary Table 1**, and LUAD data was organized according to **Supplementary Table 3**.

S.2.3 Results of the Transcriptome-wide Causal Inference

With both transcriptomic and genotypic data of the 528 samples, SIGNET constructed the transcriptome-wide GRN for LUAD. Out of a total of 17,305 genes passing the quality control, 7,039 genes were identified with IVs, consisting of 17,794 SNPs or SNP regions (14,581 common variants, 1,161 regions of low-frequency variants, and 2,052 regions of rare variants). The results are shown in **Supplementary Fig. 2**.

SIGNET bootstrapped 1,000 datasets and conducted transcriptome-wide causal inference of gene regulation on each of these datasets. It detected 4,079 gene regulations involving 4,904 genes in each bootstrap dataset, and 21,277 gene regulations involving 12,278 genes in over 95% of these bootstrap datasets (**Supplementary Fig. 2**). The complete list of these gene regulations is shown in **Supplementary Table 10**.

S.2.4 Annotation and Validation of the Constructed GRN

We investigated the GRN detected in every bootstrap data set and identified the 5th-largest subnetwork (**Supplementary Fig. 1d),** which involves 35 genes including 10 transcription factors and shares 25 genes with the largest subnetwork constructed from GTEx data for healthy lung tissues (**Fig. 2d**). Validation in STRING[6] shows that this set of genes is enriched in 15 human KEGG pathways ($p < 10^{-5}$) with the top ten shown in **Supplementary Fig. 1f**. These pathways include the IL-17 signaling pathway ($p = 1.53 \times 10^{-15}$) and the TNF signaling pathway ($p = 5.94 \times 10^{-13}$), both of which play an important role in the immune response (**Supplementary Table 11**). As highlighted in **Supplementary Fig. 1d-e**, STRING also shows rich connections between genes, evidenced via text mining, experiments, database, and co-expression with score over 0.8. Note that our constructed GRN further reveals the causal regulation between these genes.

We also validated the same set of genes using IPA[7] but restricting to human lung tissues and identified 12 Ingenuity canonical pathways (**Supplementary Table 12**) enriched with these genes ($p \leq 10^{-5}$), with top ten pathways shown in **Supplementary Fig. 2a**. Note that four of the top ten pathways are related to IL-17, as both pathways on cytokine production are on the differential regulation of cytokine production between IL-17A and IL-17F. IPA also reports that this set of genes is significantly associated with 33 types of diseases and functions ($p \leq 10^{-5}$). For the five types reported in **Fig. 3b**, we showed their significance in **Supplementary Fig. 2b** with details in **Supplementary Table 13**. In fact,

there are 25 genes associated with neurological disease, 24 genes with infectious disease, and 33 genes with organismal injury and abnormalities.

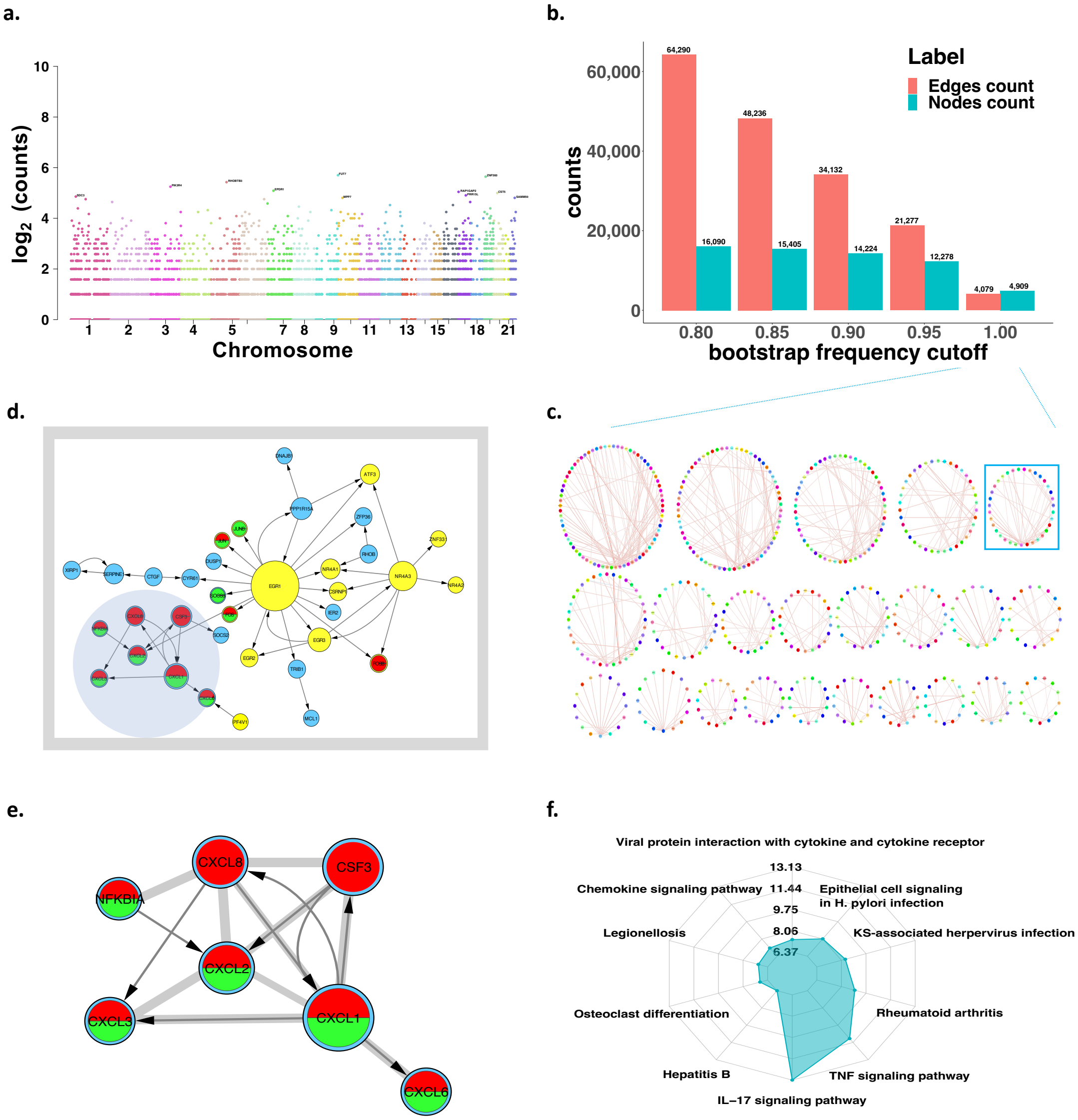

Supplementary Figure 1. Results of analyzing LUAD data in TCGA. a, Manhattan plot of numbers of IVs across all chromosomes. **b**, Histogram of numbers of edges and nodes with respect to different bootstrap frequency cutoffs. **c**, Circular plot of the largest subnetwork, with darker color indicates larger size of regulatory effects. **d**, The 5th largest subnetwork, with transcription factors shown in yellow and node sizes proportional to node degrees. **e**, Highlight of gene regulations shaded in d with gray connections verified by STRING, which also identified genes in red and green respectively enriched in IL-17 and TNF signaling pathways. **f**, Radar plot of the ten KEGG pathways in which the subnetwork in d is enriched the most, with IL-17 and TNF signaling pathways the top two.

a.

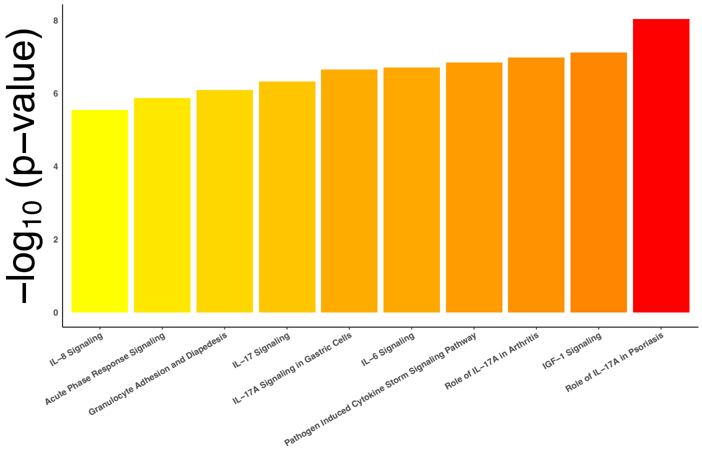

b.

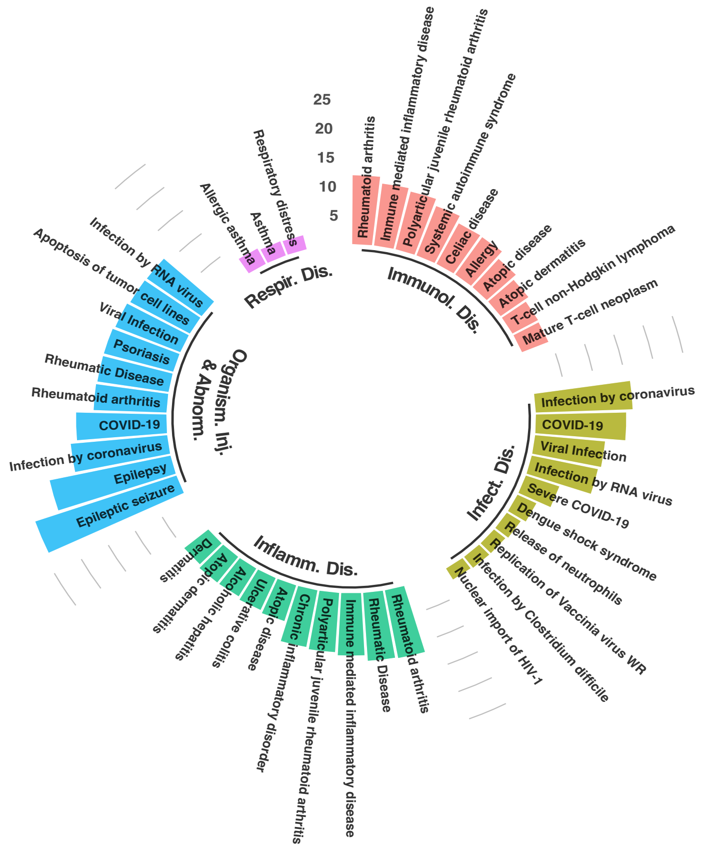

Supplementary Figure 2. IPA validation of the 5th largest subnetwork constructed from LUAD data in TCGA. a, Top ten significant Ingenuity canonical pathways. **b**, The five types of diseases and functions that match the healthy lung result in **Fig. 3b** identified by IPA, with each type shown the at most top ten significant diseases/functions.

Supplementary Table 1. Path Settings for installing SIGNET

Path	Usage
path_to_start	path inside which SIGNET is installed
path_to_signet	path to SIGNET
path_to_sif	path to the singularity container for SIGNET
path_to_cluster_signet	cluster path to SIGNET
path_to_cluster_sif	cluster path to the singularity container for SIGNET

Supplementary Table 2. Path settings for analyzing GTEx data.

**Path**	**Usage**
path_to_vcf0	path to unphased genotypic data in vcf format
path_to_vcf	path to phased genotypic data in vcf format
path_to_read	path to gene count read data
path_to_tpm	path to gene count TPM data
path_to_pheno	path to phenotype file
path_to_gtf	path to collapsed genecode gtf file

Supplementary Table 3. Path settings for analyzing TCGA data.

**Path**	**Usage**
path_to_ped	path to the SNP ped file
path_to_map	path to the SNP map file
path_to_gmap	path to the recombination map file for imputation
path_to_ref	path to the reference file for imputation
path_to_clinical	path to the file with clinical information
path_to_count	path to count data from UCSC
path_to_pmap	path to collapsed genecode gtf file

Supplementary Table 4. Instrumental variables for healthy lung tissues (Separate file)

This table lists all IVs ($p < 0.05$) that were identified for healthy lung tissues.

Supplementary Table 5. Detailed gene regulations for healthy lung tissues (Separate file)

This table details the GRN constructed for healthy lung tissues, with gene regulations detected in at least 80% of the 1,000 bootstrap datasets.

Supplementary Table 6. Enriched KEGG pathways or processes identified for healthy lung tissues by STRING (Separate file)

This table lists the KEGG pathways or Processes ($p < 10^{-5}$) that are enriched with genes from the top five subnetworks constructed for healthy lung tissues. The pathways and processes were identified by STRING, and the top five subnetworks were consistently identified in each of the 1,000 bootstrap datasets for healthy lung tissues.

Supplementary Table 7. Ingenuity canonical pathways identified for healthy lung tissues by IPA (Separate file)

This table lists the Ingenuity canonical pathways ($p < 10^{-5}$) which are enriched with genes from the top five subnetworks constructed for healthy lung tissues. The pathways were identified by IPA, and the top five subnetworks were consistently identified in each of the 1,000 bootstrap data sets for healthy lung tissues.

Supplementary Table 8. Diseases and functions identified for healthy lung tissues by IPA (Separate file)

This table lists the diseases and functions ($p < 10^{-5}$) which are significantly associated with genes from the top five subnetworks constructed for healthy lung tissues. The diseases and functions were identified by IPA, and the top five subnetworks were consistently identified in each of the 1,000 bootstrap datasets for healthy lung tissues.

Supplementary Table 9. Instrumental variables for LUAD (Separate file)

This table lists all IVs ($p < 0.05$) that were identified for LUAD.

Supplementary Table 10. Detailed gene regulations for LUAD (Separate file)

This table details the GRN constructed for LUAD, with gene regulations detected in at least 80% of the 1,000 bootstrap datasets.

Supplementary Table 11. Enriched KEGG pathways or processes identified for LUAD (Separate file)

This table lists the KEGG pathways or processes ($p < 10^{-5}$) which are enriched with genes from the top five subnetworks constructed for LUAD. The pathways and processes were identified by STRING, and the top five subnetworks were consistently identified in each of the 1,000 bootstrap datasets for LUAD.

Supplementary Table 12. Ingenuity canonical pathways identified for LUAD by IPA (Separate file)

This table lists the Ingenuity canonical pathways ($p < 10^{-5}$) which are enriched with genes from the top five subnetworks constructed for LUAD. The pathways were identified by IPA, and the top five subnetworks were consistently identified in each of the 1,000 bootstrap data sets for LUAD.

Supplementary Table 13. Diseases and functions identified for LUAD by IPA (Separate file)

This table lists the diseases and functions ($p < 10^{-5}$) which are significantly associated with genes from the top five subnetworks constructed for LUAD. The diseases and functions were identified by IPA, and the top five subnetworks were consistently identified in each of the 1,000 bootstrap datasets for LUAD.

Supplementary File 1. Interactive visualization of the largest subnetwork constructed for healthy lung tissues (Separate file)

The HTML file generated by SIGNET shows the largest subnetwork constructed for healthy lung tissues with regulations consistently identified in each of the 1,000 bootstrap datasets. It also includes gene enrichment results obtained from STRING, which can be viewed by hovering the cursor over and clicking on genes, or selecting a pathway listed in the top-left corner. Transcription factors are highlighted in yellow, and the node size indicates the number of regulations the corresponding gene is involved with. A triangle arrow indicates an up-regulation and a circular arrow indicates a down-regulation. Users can reposition the nodes by dragging them.

Supplementary File 2. Interactive visualization of the largest subnetwork constructed for LUAD (Separate file)

This HTML file generated by SIGNET shows the largest subnetwork constructed for LUAD with regulations consistently identified in each of the 1,000 bootstrap datasets. It also includes gene enrichment results obtained from STRING, which can be viewed by hovering over and clicking on genes, or selecting a pathway listed in the top-left corner. Transcription factors are highlighted in yellow, and the node size indicates the number of regulations the corresponding gene is involved with. A triangle arrow indicates an up-regulation and a circular arrow indicates a down-regulation. Users can reposition the nodes by dragging them.

Supplementary Codes 1. Codes for Analyzing GTEx Lung Data

```
# Download SIGNET
cd path/to/start
git clone https://github.com/signet4grn/SIGNET.git

# Environment configuration should execute every time a new session starts
cd dir
export PATH=path_to_signet:$PATH
export SINGULARITYENV_APPEND_PATH=path_to_signet
export SINGULARITY_BIND= ~/

# Set cohort to GTEx
singularity exec path_to_sif signet -s --cohort GTEx
singularity exec path_to_sif signet -s --tissue Lung

# Genotype Preprocessing
(echo y | nohup singularity exec path_to_sif signet -g \
            --vcf0 path_to_vcf0 \
            --vcf path_to_vcf \
            --read path_to_read \
            --anno path_to_anno \
             --tissue Lung > \
geno.out) &

# Gexp Preprocessing
(echo y | nohup singularity path_to_sif  signet -t  \
        --read path_to_read \
        --tpm  path_to_tpm \
        --gtf  path_to_gtf > \
        gexp.out) &

# Adjust for covariates
singularity exec path_to_sif signet -s \
--anno path_to_anno
singularity exec path_to_sif signet -s \
--gtf.file path_to_gtf
printf "Y\n3" | singularity exec path_to_sif signet -a \
--pheno path_to_pheno > adj.out &

# cis-eQTL
(echo Y | nohup singularity exec path_to_sif \
signet -c --gexp res/resa/signet_gexp_rmpc.data \
            --gexp.withpc res/resa/signet_gexp.data \
            --geno res/resg/signet_clean_Genotype_repNA.data \
            --map res/resg/signet_snps.map \
            --maf res/resg/signet_snps.maf \
            --gene_pos res/resa/signet_gene_pos > \
            cis.out) &

# Computing in research cluster
```

```
cd  path_to_cluster_signet
export PATH=path_to_cluster_signet/SIGNET
export SINGULARITYENV_APPEND_PATH=path_to_cluster_signet
export SINGULARITY_BIND=~/

# Network Analysis
(echo y | nohup signet -n \
            --nboots 1000 \
            --cor 0.3 \
            --ncores 50 \
            > network.out) &

# Visualization
echo y | singularity exec path_to_signet signet -v \
           --freq 1 \
           --ntop 3
```

Supplementary Codes 2. Codes for Analyzing LUAD Data in TCGA

```
cd path_to_start
git clone https://github.com/signet4grn/SIGNET.git

# Should execute every time a new session starts
cd path_to_start
export PATH=path_to_signet:$PATH
export SINGULARITYENV_APPEND_PATH=path_to_signet
export SINGULARITY_BIND=~/

# Gene Expression Preprocessing
singularity exec path_to_sif signet -t \
                --gexp.file path_to_count \
                --pmap.file path_to_pmap

# Genotype Preprocessing
(echo y | nohup singularity exec path_to_sif signet -g \
                       --ped path_to_ped \
                       --map path_to_map \
                       --gmap path_to_gmap \
                       --ref path_to_ref  > geno.out) &

# Adjust for covariates (10PCs adjusted)
singularity exec path_to_sif signet -a \
               --c path_to_clinical

# cis-eQTL
(echo y | nohup singularity exec path_to_sif signet -c > \
cis.out)  &

# Network analysis
## In research cluster
cd path_to_cluster_signet
export PATH=path_to_cluster_signet:$PATH
export SINGULARITYENV_APPEND_PATH=path_to_cluster_signet
export SINGULARITY_BIND=~/
(echo y | nohup signet -n \
                     --nboots 1000 \
                     --cor 0.3 > network.out) &

# Visualize
echo y | singularity exec path_to_sif signet -v \
                         --freq 1 \
                         --ntop 10 > netvis.out


echo y | singularity exec path_to_sif signet -v \
                         --freq 1 \
                         --ntop 3
```

References

1. Consortium, G. The GTEx Consortium atlas of genetic regulatory effects across human tissues. *Science* ***369**,* 1318–1330 (2020)
2. Purcell, S. *et al.* PLINK: A tool set for whole-genome association and population-based linkage analyses. *Am. J. Hum. Genet.* **81**, 559–575 (2007).
3. Weinstein, J. N. *et al.* The Cancer Genome Atlas Pan-cancer analysis project. *Nat. Genet.* **45**, 1113–1120 (2013).
4. Li, H. & Durbin, R. Fast and accurate short read alignment with Burrows-Wheeler transform. *Bioinformatics* **25**, 1754–1760 (2009).
5. Kim, S. *et al.* Strelka2: fast and accurate calling of germline and somatic variants. *Nat. Methods* **15**, 591–594 (2018).
6. Szklarczyk, D. *et al.* STRING v11: Protein-protein association networks with increased coverage, supporting functional discovery in genome-wide experimental datasets. *Nucleic Acids Res.* **47**, D607–D613 (2019).
7. Krämer, A., Green, J., Pollard, J. & Tugendreich, S. Causal analysis approaches in Ingenuity Pathway Analysis. *Bioinformatics* **30**, 523–530 (2014).
